# Effects of Running in Minimal and Conventional Footwear on Medial Tibiofemoral Cartilage Failure Probability in Habitual and Non-Habitual Users

**DOI:** 10.3390/jcm11247335

**Published:** 2022-12-09

**Authors:** Jonathan Sinclair, Guohao Huang, Paul John Taylor, Nachiappan Chockalingam, Yifang Fan

**Affiliations:** 1 Research Centre for Applied Sport, Physical Activity and Performance, School of Sport & Health Sciences, Faculty of Allied Health and Wellbeing, University of Central Lancashire, Preston PR1 2HE, Lancashire, UK; 2 Foot Research Laboratory, Key Laboratory of Sport and Health Science of Fujian Province, School of Physical Education and Sport Science, Fujian Normal University, Fuzhou 350117, China; 3 School of Psychology & Computer Sciences, Faculty of Science and Technology, University of Central Lancashire, Preston PR1 2HE, Lancashire, UK; 4 School of Life Sciences and Education, Staffordshire University, Stoke on Trent ST4 2DE, UK

**Keywords:** running, medial tibiofemoral compartment, musculoskeletal simulation, minimal footwear, osteoarthritis

## Abstract

This study examined the effects of minimal and conventional running footwear on medial tibiofemoral cartilage mechanics and longitudinal failure probability. The current investigation examined twenty males who habitually ran in minimal footwear and 20 males who habitually ran in conventional footwear. Kinematic data during overground running were collected using a motion-capture system and ground reaction forces using a force plate. Medial tibiofemoral loading was examined using musculoskeletal simulation and cartilage failure probability via probabilistic modelling. In habitual minimal footwear users, peak medial tibiofemoral cartilage force, stress and strain were significantly greater in conventional (force = 7.43 BW, stress = 5.12 MPa and strain = 0.30), compared to minimal footwear (force = 7.11 BW, stress 4.65 MPa and strain = 0.28), though no significant differences in these parameters were evident in non-habitual minimal footwear users (conventional: force = 7.50 BW, stress = 5.05 MPa and strain = 0.30; minimal: force = 7.40 BW, stress = 4.77 MPa and strain = 0.29). However, in both habitual and non-habitual minimal footwear users, the probability of medial tibiofemoral cartilage failure was significantly greater in conventional (habitual = 47.19% and non-habitual = 50.00%) compared to minimal footwear (habitual = 33.18% and non-habitual = 32.81%) users. The observations from this investigation show that compared to minimal footwear, conventional footwear appears to have a negative influence on medial tibiofemoral cartilage health.

## 1. Introduction

Running is an extremely popular recreational activity and represents one of the most popular aerobic exercise activities [1]. Regular engagement with this exercise modality produces a variety of physiological and psychological benefits [2]. However, despite the physical benefits, it is associated with a very high prevalence of chronic pathologies [3]. Such pathologies are a significant barrier to training adherence and impose a substantial fiscal burden [4]. Importantly, the knee has been shown to be the most frequently injured musculoskeletal structure in runners, accounting for as many as 50% of all pathologies [3].

The knee plays an important role in load bearing during running, walking and other daily activities [5]. However, knee osteoarthritis (OA) is the predominant source of global musculoskeletal disability [6] and imposes an extremely high global healthcare burden [7]. Knee OA is a progressive disease of articular cartilage, depicted by degeneration of the cartilage within the knee joint [8]. Patients with knee OA suffer from enduring pain and lack of joint function [9]. Causal factors in the initiation and progression of OA remain contentious, but high mechanical loads imposed onto the cartilage itself during everyday activities have been shown to be important aetiological factors [10]. Knee OA is most commonly observed at the medial aspect of the tibiofemoral joint [11], as during the majority of everyday activities, knee loads predominantly pass through the medial compartment [12].

Running footwear may play an important role in chronic injury prevention [13] and over the past 40 years significant innovations in running footwear technology have been made [14]. In recent years, minimal footwear, which does not feature high levels of cushioning or motion control features that are commonplace in conventional running shoes, has received considerable research attention [14,15]. Randomized controlled trials have importantly shown that long-term use of minimal footwear improved pain symptoms in elderly patients with existing knee OA [16] and prevented knee pain in runners preparing for a 10-kilometre event [17]. Several observational analyses have also examined running kinetics and kinematics in minimal and conventional footwear, although only limited analyses have examined their effects on the loads borne by the medial tibiofemoral joint. Sinclair et al. [15] showed that minimal footwear significantly increased the magnitude of the knee adduction moment, which is utilized as a quasi-measure of medial knee contact loading [18]. Finally, Sinclair et al. [19] using a musculoskeletal simulation-based approach, found that minimal footwear was associated with significant increases in medial tibiofemoral loading during early stance, compared to conventional footwear.

However, previous observational biomechanical analyses examining medial tibiofemoral loading have been undertaken using runners who do not habitually utilize minimal footwear. Tam et al. [20] proposed that investigations of non-habitual minimal footwear users are not representative, and that a period of accommodation is necessary to adapt to such a novel footwear stimulus. Therefore, additional investigation of minimal and conventional footwear is required in runners who habitually wear minimal and conventional footwear. Furthermore, although advances in musculoskeletal simulation modelling have been made [21]. There remain considerable challenges in examining the effects of different footwear and joint-loading scenarios on the initiation and time course of knee OA. Therefore, computational probabilistic modelling of cartilage mechanics may be valuable for quantifying the probability of OA over a lifetime of cyclic loading in runners adopting different footwear modalities [22]. Such approaches have not yet been utilized to examine differences in medial tibiofemoral cartilage failure probability between minimal and conventional running shoes in those who run habitually in minimal footwear.

The aim of this study is to investigate the effects of minimal and conventional running footwear on medial tibiofemoral cartilage mechanics and lifetime failure probability via a collective musculoskeletal simulation and computational-modeling-based approach in both habitual and non-habitual minimal footwear users. This investigation hypothesizes that minimal footwear will increase medial tibiofemoral cartilage loading mechanics and also lifetime failure probability in relation to conventional footwear in both habitual and non-habitual minimal footwear users.

## 2. Materials and Methods

### 2.1. Participants

Twenty males who habitually ran using conventional footwear and twenty males who habitually used minimal footwear (henceforth termed habitual minimal footwear users) volunteered for the current investigation. For inclusion into this investigation, participants were required to complete a minimum of 35 km per week of training and be between the ages of 18 and 40. For inclusion into the habitual minimal footwear group, participants were required to have been training exclusively in minimal footwear for a minimum period of 24 months in footwear scoring ≥75 on the minimalist index [23,24,25,26]. Using data from our previous work [19], with a mean ± SD difference in peak medial tibiofemoral force of 0.69 ± 0.76 between conditions, it was determined that in order to achieve α = 5% and β = 0.80, 20 runners would be needed in both non-habitual and habitual minimal footwear groups. All participants were free from pathology at the time of data collection and provided written informed consent, in accordance with the principles outlined in the Declaration of Helsinki. The procedure utilized for this investigation was approved by a university ethics committee (REF 637).

### 2.2. Procedure

Three-dimensional retroreflective marker data were collected using a motion-capture system with 8 cameras (Qualisys Medical AB, Gothenburg, Sweden) using a capture rate of 250 Hz. The motion-capture system was calibrated prior to the commencement of each data collection session. Ground reaction forces (GRF) were collected using a piezoelectric force plate (Kistler Instruments Ltd., Winterthur, Switzerland) which sampled at 1000 Hz. Kinematic and GRF data were collected in a synchronous manner.

**Figure 1:**
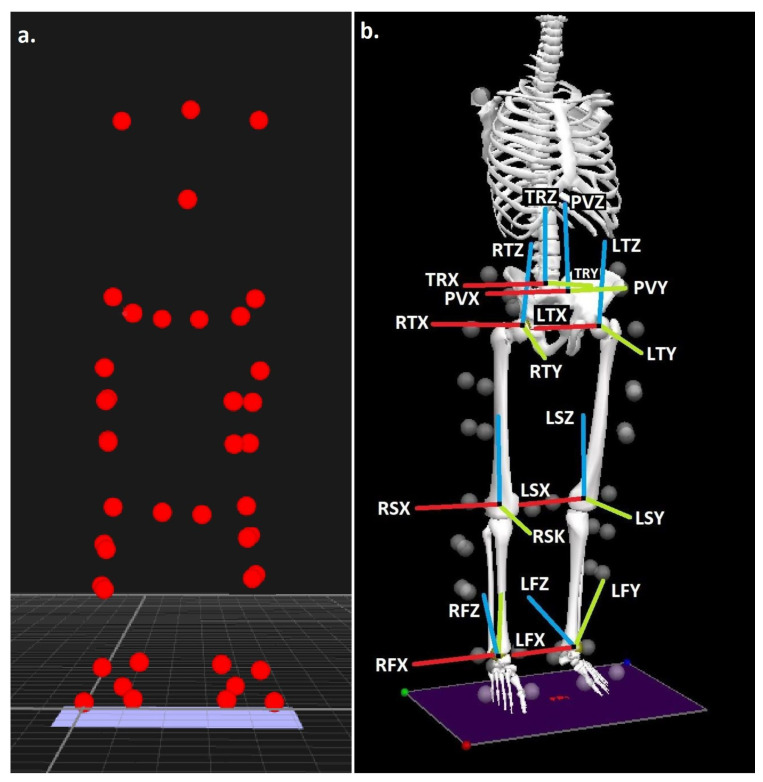
(**a**) Experimental marker locations and (**b**) trunk, pelvis, thigh, shank and foot segments, with segment co-ordinate system axes (R = right and L = left), (TR = trunk, P = pelvis, T = thigh, S = shank and F = foot), (X = sagittal, Y = coronal and Z = transverse planes).

Body segments were modelled in 6 degrees of freedom using the calibrated anatomical systems (CAST) technique [24], using a marker/model configuration utilized previously to quantify the biomechanics of both walking and running [19,25] (Figure 1a,b). Intra-rater reliability for the individual responsible for positioning of the anatomical markers has been shown to be excellent (ICC ≥ 0.931) [26]. The centres of the ankle and knee joints were the midpoints between the malleoli and femoral epicondyle markers [27,28] and the hip joint centre was established via a regression approach that utilizes the positions of the anterior superior iliac spine markers [29].

#### 2.2.1. Walking

As outlined in the study aims, this investigation is principally interested in examining the effects of minimal and conventional footwear during running. However, both running and walking commutatively comprise the daily locomotion distance that contributes to longitudinal joint loading and ultimately cartilage failure probability in runners [22]. Therefore, in order to quantify cartilage failure as accurately as possible, participants were tested during walking only in the conventional footwear, as none of the habitual minimal footwear users indicated that they performed their everyday walking using such footwear. Walking data are presented in Supplementary Materials Section S4. Participants walked across the laboratory, striking the force platform with their right (dominant) foot. A self-selected walking velocity was adopted to improve ecological validity and allow the true longitudinal effects on medial tibiofemoral cartilage to be examined, as a fixed velocity would not be feasible in the real world. Five successful trials were collected. For a trial to be successful, the foot had to make full contact with the force platform with no evidence of gait modifications due to the experimental conditions.

#### 2.2.2. Running

Both participant groups ran across the laboratory in both minimal and conventional running footwear, striking the force platform with their right (dominant) foot. Once again, to improve ecological validity, a self-selected velocity was adopted. Five successful trials were collected in each footwear condition which were tested in a counterbalanced order, using the same criteria for success as described in the aforementioned walking section.

### 2.3. Experimental Footwear

**Figure 2:**
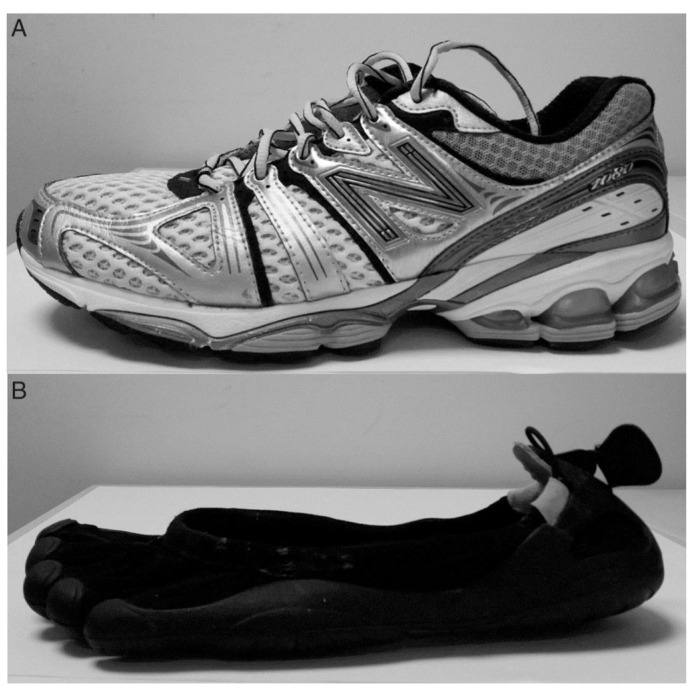
Experimental footwear (**A**) = conventional running shoes, (**B**) = minimal.

The footwear used during this study consisted of New Balance, 1080 (New Balance, Boston, MA, USA; termed conventional) and Vibram Five-Fingers (Vibram, Albizzate, Italy; henceforth termed minimal) (Figure 2; Table 1).

### 2.4. Processing

Four total data-processing phases were undertaken that used the walking and running biomechanical data to estimate long-term medial tibiofemoral cartilage failure probability: (1) spatiotemporal variables, (2) joint contact forces, (3) stress/strain and (4) medial tibiofemoral cartilage failure probabilistic modelling.

#### 2.4.1. Data Processing

Dynamic walking and running trials were digitized using Qualisys Track Manager (Qualisys Medical AB, Gothenburg, Sweden) in order to identify anatomical and tracking markers, and then exported as C3D files to Visual 3D (C-motion, Germantown, MD, USA). All data were linearly normalized to 100% of the stance phase, which was delineated as the duration over which 20 N or greater of vertical GRF was applied to the force plate [30]. GRF data and marker trajectories were smoothed with cut-off frequencies of 50 Hz and 6 Hz for walking and 50 Hz and 12 Hz for running, respectively, using a low-pass Butterworth 4th-order zero-lag filter. Kinetic and kinematic cut-off frequencies were obtained using residual analyses [31].

#### 2.4.2. Spatiotemporal Variables

Both walking and running velocities were quantified within Visual 3D, using the linear velocity of the model centre of mass in the anterior direction [32] and stride lengths were determined by calculating the difference in the anterior position of the foot’s centre of mass at footstrike between initial and subsequent ipsilateral foot contacts [32]. During running, cadence (steps/min) was also extracted by calculating the time between initial and subsequent ipsilateral foot contacts and determining the number of footfalls that would be achieved per minute.

#### 2.4.3. Joint Contact Forces

Stance phase data during both walking and running were exported from Visual 3D into OpenSim software version 3.3 (Simtk.org). An existing musculoskeletal model [33] was first scaled to account for each participant’s anthropometrics. This model has been adopted previously in order to quantify medial and lateral tibiofemoral loading during both walking and running activities [25,32]. A residual reduction algorithm [21] was adopted first and then muscle forces were quantified using a static optimization process within OpenSim, as described by Steele et al. [34]. Following static optimization, the peak normalized medial tibiofemoral joint force (BW) was calculated using a joint reaction analysis function in OpenSim [21]. Finally, the medial tibiofemoral cumulative load was quantified by dividing the average tibiofemoral contact force by the stride length in accordance with Miller and Krupenevich [22]. The peak lateral tibiofemoral force (BW) was also quantified, and although not included in the probabilistic model, is presented in the appendices (Supplementary Materials Section S5) for information regarding potential redistribution of tibiofemoral forces. Pilot analyses from our laboratory have shown high reliability (ICC ≥ 0.901) and minimal detectable differences (MDC) of 0.27 and 0.61 BW for the peak medial tibiofemoral force during running and walking, respectively (Supplementary Materials Section S1).

Furthermore, the peak forces (BW) during the stance phase for the major muscles crossing the knee joint (rectus femoris, vastus medialis, vastus lateralis, vastus intermedius, semitendinosus, semimembranosus, biceps femoris long head, biceps femoris short head, lateral gastrocnemius, medial gastrocnemius, sartorius and gracilis) were quantified and the impulses (BW·ms) of these muscles during the stance phase were also extracted using a trapezoidal function [19].

#### 2.4.4. Stress/Strain

The aforementioned medial tibiofemoral contact forces during the stance phase of walking and running were utilized as inputs into a tibiofemoral contact model to determine the peak stress and strain indices in the tibiofemoral cartilage using MATLAB code adapted from Miller and Krupenevich [22]. Pilot analyses from our laboratory have shown high reliability (ICC ≥ 0.898) and MDCs of 0.01 and 0.02 during running and 0.16 and 0.35 MPa during walking for peak medial tibiofemoral stress and strain (S1). The medial tibiofemoral contact mechanics model was based on that developed and described by Nuño and Ahmed [35]. Briefly, medial tibiofemoral contact stresses (σ) and strains (ε) were calculated based on Equations (1) and (2).

σ = −Mean tibiofemoral cartilage modulus × Log (1 − ε)(1)

ε = Cartilage element compression/modelled cartilage height(2)

The medial femoral condyle was delineated as two convex curves representing its anterior and posterior components from a sagittal perspective and a single curve from a coronal posterior viewpoint, whereas the tibial plateau was modelled conversely as a concave curve. The radius of the tibial arc in the frontal plane was assumed to be 21 mm and\those of the anterior and posterior aspects of the femoral arc in the sagittal plane were modelled as 35.0 mm and 18.9 mm, respectively. The tibial segment was assumed to be held in a fixed position in space but in accordance with the Nuño and Ahmed [35] model, the femur featured two variable parameters: the elevation of the knee flexion axis with respect to the tibia and the angle of flexion itself. The articular cartilage within the tibiofemoral joint itself was depicted as a series of elements upon the tibial plateau, with an unloaded height of 5.0 mm [36]. The modelled articular cartilage elements themselves were assumed to exhibit a nonlinear elastic stress–strain relationship [37].

Peak tibiofemoral contact stresses (MPa) and strains were calculated as a function of the loaded cartilage modulus at this joint, the magnitude of the compression of the modelled contact elements and the modelled number of contact elements, which was considered to be 7326 accounting for a modelled distance between elements of 0.5 mm and the aforementioned tibial and femoral arc radii. The tibiofemoral cartilage moduli differed as a function of the location of the loaded contact elements as some were concealed by the medial meniscus. The moduli included in the model for the femoral cartilage, unconcealed tibial cartilage and concealed tibial cartilage, were 8.6, 4.0 and 10.1 MPa [38]. The medial meniscus modulus was considered to be 1.3 MPa and the meniscus itself was modelled to conceal 46% of the tibial plateau [39,40]. Owing to the increased rates of loading during running, in accordance with Miller and Krupenevich [22], the cartilage elastic moduli, unloaded cartilage height and anterior and posterior aspects of the femoral arc in the sagittal plane were increased by a factor of 1.5 times the standard deviation of these values as summarized by Miller and Krupenevich [22]. The cartilage and menisci were delineated with a Poisson’s ratio of 0.45 [41].

The knee flexion angle input into the model was the angle at which peak medial tibiofemoral force occurred. The vertical height of the knee flexion axis was initially set so that the cartilage was unloaded and then incrementally decreased until the contact force matched that from the input medial tibiofemoral contact force. With distinct modelled radius distances for the anterior and posterior aspects of the medial femur, the area of cartilage under load differed as a function of the knee flexion angle [42]. Translational mechanics of the knee joint were not included in the model as the medial aspect of the femoral condyle typically remains close to the center of the tibial plateau despite changes in the knee flexion angle [43].

#### 2.4.5. Medial Tibiofemoral Cartilage Failure Probabilistic Modelling

The cartilage failure probabilistic model in the current investigation is adapted from Miller and Krupenevich [25] and the reader is directed to this open access publication for images of the modelled anatomy. Pilot sensitivity analyses from our laboratory were performed to examine the sensitivity of the two key outcome parameters, peak medial tibiofemoral strain and probability of cartilage failure, to variations within feasible ranges of each model input parameter in isolation, holding the others at their modelled (i.e., described below) values (Supplementary Materials Section S2). In accordance with Miller and Krupenevich [22], cartilage failure at the medial aspect of the tibiofemoral joint was delineated as macroscopic plastic deformation typical of early-stage osteoarthritic degeneration [44]. As the median age of knee OA diagnosis is 55 years [45], the probability of cartilage failure was calculated over a period of 37 years, from skeletal maturity aged 18 until 55 years of age [46]. Probability of failure was calculated using probabilistic models of tissue damage and repair [47,48,49], with cartilage strain as its equivalent for damage.

The probability of cartilage failure at the specified time (i.e., 37 years) was expressed as a cumulative function of the modelled cartilage experiencing loading cycles over a specific distance each day with the stride length from the initial kinematic processing using Equation (3).

Probability of cartilage failure = 1 − Exp − [(Volume of stressed cartilage/Reference stressed cartilage volume) (time/time until failure) Weibull exponent/Power law exponent](3)

In Equation (3) the reference stressed cartilage (78.5 mm^3^), the Weibull exponent (14.3) and the power law exponent (12.9) were all constants in the cartilage failure probability model. Time until failure was calculated using Equation (4).

Time to failure = (Power law coefficient × Stride length/Distance per day) (Weibull coefficient × ε) Power law exponent(4)

The Weibull coefficient (1.03), power law coefficient (1.0), Weibull exponent (14.3) and power law exponent (12.9) were all constants in the time-until-failure model. Values for the power law and Weibull parameters were obtained from Miller and Krupenevich [22], who fit a power law for cycles to failure to the scattered fatigue modelling data from Riemenschneider et al. [50]. The reference stressed cartilage (78.5 mm^3^) is the data from which the other parameters were determined. In Equation (4), in accordance with Miller and Krupenevich [22], the time until failure is the scattered fatigue distribution’s scale parameter, i.e., the time at which 63.2% of cases would fail when experiencing the modelled daily number and magnitude of cartilage strains per day. Distance travelled per day was inputted into the model as 8.0 km per day throughout, which was modelled as participants completing 3.0 km per day of walking using data inputted from walking collected from both groups and 5 km per day of running, using data inputted from both groups running in each footwear condition.

Equation (3) represents the probability of failure of cartilage in vitro. As living cartilage is able to recover partially from strain-imposed damage [51], the probability of medial tibiofemoral cartilage repair was therefore modelled using Equation (5).

Probability of repair = 1 − Exp − [ − (time/time until repair) Cartilage repair exponent](5)

In Equation (5), time until repair (5.0 years) and cartilage repair exponent (5.2) were constants extracted from Miller and Krupenevich [22] and the time until repair was modelled as the duration after which repair was expected in 63.2% of damage cases.

Repair itself was integrated into equation number 3 in accordance with Miller and Krupenevich [22] by deriving a probability density function that determines the instantaneous probability of failure at a given time point in time. This process is outlined in Equation (6) below.

Probability density function = (Volume of stressed cartilage × Weibull exponent/power law exponent × Reference stressed cartilage volume × time until failure) (time/time until failure) Weibull exponent/Power law exponent-1 exp [−(Volume of stressed cartilage/Reference stressed cartilage volume) (time/time until failure) Weibull exponent/Power law exponent](6)

The probability density function is then multiplied by the collective likelihood that repair has not yet taken place (i.e., 1-Probability of repair) and then time integrated to determine the probability of failure taking into account repair. This process is outlined in Equation (7) below.

Probability of failure with repair = ∫ (time 0) [Probability density function × (1 − Probability of repair)] modelled distance between contact elements × time.(7)

### 2.5. Statistical Analyses

Means and standard deviations (SD) were calculated for each experimental variable. Differences in participant characteristics (age, stature, mass, body mass index (BMI) and weekly running volume) and spatiotemporal parameters, medial tibiofemoral forces and stress/strain parameters during walking between the non-habitual and habitual minimal footwear groups were examined using between-groups linear mixed-effects models with group modelled as a fixed factor. During running medial tibiofemoral forces and stress/strain parameters were contrasted using two separate comparisons: (1) minimal footwear vs. conventional footwear in non-habitual minimal footwear users and (2) minimal footwear vs. conventional footwear in habitual minimal footwear users. To accomplish this, within-subjects linear mixed-effects models were adopted utilizing the compound symmetry method, with footwear modelled as a fixed factor. Finally, to compare the medial tibiofemoral cartilage failure-probability indices over the aforementioned period of 37 years, the same two comparisons were made whilst also incorporating the modelled data during walking from each group. Linear mixed-effects models were all adopted with participants representing the random intercept in all cases whilst also using the restricted maximum-likelihood method. As the current investigation utilized a self-selected running velocity, its effects on the comparisons between footwear of peak medial tibiofemoral force, peak medial tibiofemoral stress and peak medial tibiofemoral strain were examined. First, linear regression models were undertaken with running velocity as the predictor variable and the aforementioned joint loading indices as dependent variables. Second, the two separate comparisons described above were repeated, only with running velocity included into the linear mixed-effects model as a continuous co-variate. Statistical significance for all analyses was accepted at the *p* < 0.05 level and all statistical analyses were conducted using SPSS v27 (IBM, SPSS, Armonk, NY, USA).

## 3. Results

### 3.1. Participant Characteristics

Comparisons between groups for age, mass, stature, BMI and weekly running volume were non-significant (*p* = 0.24–0.95) (Table 2).

### 3.2. Running Analyses

Spatiotemporal, joint contact forces, stress/strain parameters during running and medial tibiofemoral cartilage failure probabilistic indices are presented in Table 3, Table 4 and Table 5. Muscle force, walking data, lateral tibiofemoral forces and co-variate/regression analyses are presented in Supplementary Materials Sections S3–S6, respectively.

#### 3.2.1. Spatiotemporal Variables

In the habitual minimal footwear group, both running velocity (*b* = 0.07 _(95% CI = 0.03–0.10)_, t = 4.03, *p* = 0.001) and stride length (*b* = 0.12 _(95% CI = 0.01–0.09),_ t = 6.94, *p* < 0.001) were significantly greater in conventional than in minimal footwear. Furthermore, in both the habitual (*b* = 6.57 _(95% CI = 3.17–9.97)_, t = 4.04, *p* = 0.001) and non-habitual minimal footwear groups (*b* = 7.53 _(95% CI = 3.39–11.67)_, t = 3.81, *p* = 0.001), cadence was significantly greater in minimal compared to conventional footwear (Table 3).

**Table 3:** Spatiotemporal variables.

	Non-Habitual				Habitual				
	Minimal		Conventional		Minimal		Conventional		
	Mean	SD	Mean	SD	Mean	SD	Mean	SD	
Velocity (m/s)	3.77	0.39	3.68	0.44	3.36	0.56	3.43	0.53	**A**
Stride length (m)	2.80	0.30	2.82	0.28	2.46	0.28	2.58	0.34	**A**
Cadence (steps/min)	157.86	13.22	150.33	11.02	152.36	10.77	145.79	8.25	**A, B**

Notes: **A** = significant difference between minimal and conventional footwear in habitual group. **B** = significant difference between minimal and conventional footwear in non-habitual group.

#### 3.2.2. Joint Contact Forces

In the habitual minimal footwear group, peak medial tibiofemoral force (*b* = 0.32 _(95% CI = 0.23–0.87)_, t = 2.42, *p* = 0.03) was significantly greater in conventional than in minimal footwear (Table 4).

**Table 4:** Joint contact force parameters.

	Non-Habitual				Habitual				
	Minimal		Conventional		Minimal		Conventional		
	Mean	SD	Mean	SD	Mean	SD	Mean	SD	
Peak medial tibiofemoral force (BW)	7.40	0.97	7.50	0.73	7.43	1.61	7.11	1.38	**A**
Medial tibiofemoral cumulative load (BW/m)	2.59	0.33	2.61	0.41	2.97	0.58	2.80	0.59	

Notes: **A** = significant difference between minimal and conventional footwear in habitual group. SD = significant difference between habitual and non-habitual groups in conventional footwear.

#### 3.2.3. Stress/Strain

In the habitual minimal footwear group, peak medial tibiofemoral stress (*b* = 0.47 _(95% CI = 0.25–0.70)_, t = 4.41, *p* < 0.001) and strain (*b* = 0.02 _(95% CI = 0.01–0.04)_, t = 4.33, *p* < 0.001) were significantly greater in conventional than in minimal footwear (Table 5).

**Table 5:** Stress/strain parameters.

	Non-Habitual				Habitual				
	Minimal		Conventional		Minimal		Conventional		
	Mean	SD	Mean	SD	Mean	SD	Mean	SD	
Peak medial tibiofemoral stress (MPa)	4.77	0.57	5.05	0.66	4.65	0.82	5.12	0.80	**A**
Peak medial tibiofemoral strain	0.29	0.03	0.30	0.03	0.28	0.04	0.30	0.04	

Notes: **A** = significant difference between minimal and conventional footwear in habitual group.

#### 3.2.4. Medial Tibiofemoral Cartilage Failure Probabilistic Modelling

In the non-habitual minimal footwear group, the probability of failure was significantly greater (*b* = 14.00 _(95% CI = 1.52–26.49)_, t = 2.35, *p* = 0.03) in conventional than in minimal footwear. Furthermore, in the habitual minimal footwear group the probability of failure was significantly greater (*b* = 17.20 _(95% CI = 8.34–26.05)_, t = 4.06, *p* = 0.001) in conventional than in minimal footwear (Table 6).

## 4. Discussion

The aim of this study was to investigate the influence of conventional and minimal running shoes on medial tibiofemoral cartilage mechanics and lifetime failure probability in non-habitual and habitual minimal footwear users via a combined musculoskeletal simulation and computational modelling approach. This is the first examination of the effects of minimal and conventional footwear using a concurrent approach of the aforementioned techniques and may therefore provide more detailed information regarding the effects of minimal footwear on knee OA risk in runners. This investigation tested the hypotheses that minimal footwear will increase medial tibiofemoral loading indices and lifetime failure probability compared to conventional footwear in both habitual and non-habitual minimal groups.

The validity of the medial tibiofemoral joint loading components was scrutinized by comparisons to both in vivo and other biomechanical modelling information from the scientific literature. The peak medial knee forces were similar to those using both musculoskeletal modelling (2.90 BW [22]) and simulation techniques (2.73 BW [52]) using comparable walking velocities to the current investigation (1.52 and 1.50 m/s) and also to participant K8L (2.59 BW, selected as being the closest walking velocity to this investigation) from the in vivo data of Bergmann et al. [53], at a slightly slower walking velocity of 1.39 m/s. There are currently no in vivo running data at similar velocities to the current study; however, the peak medial knee loads from the current investigation are comparable to those using both musculoskeletal modelling (6.14 BW [54]) and simulation (7.57 BW [19]) at running speeds of 3.16 and 4.0 m/s. The strains experienced by the medial tibiofemoral cartilage during both walking and running were lower than those reported by Miller and Krupenevich [22] (0.23 and 0.38 at 1.52 and 2.58 m/s, respectively). However, this observation likely related to the increased cartilage modulus, cartilage thickness and tibiofemoral congruence adaptation parameters that were modelling in the current investigation to account for the increased running velocity and the tested sample of more experienced runners. Finally, in relation to the lifetime failure probability values of the medial tibiofemoral cartilage, our values are greater than those of Miller and Krupenevich [22], but the current investigation had substantially increased running volumes and velocities. Furthermore, the probability values are also greater than the epidemiological investigation of Losina et al. [45] in the general public, but in line with those of Murphy et al. [55] and more specifically those examining incidence rates in former athletes [56] and runners [57].

In opposition to our hypotheses, the current study importantly showed using musculoskeletal simulation and contact mechanical modelling that medial tibiofemoral joint forces, stresses and strains were statistically larger in conventional compared to minimal footwear in the habitual minimal footwear group. This observation opposes those of Sinclair et al. [15] using the knee adduction moment and those of Sinclair et al. [19] using musculoskeletal simulation, which both showed that, in non-habitual minimal footwear users, the medial tibiofemoral force was larger in minimal footwear. Notably, the co-variate comparisons and regression models showed less-pronounced statistical differences between footwear in the habitual minimal footwear group and running velocity as a predictor of joint forces, stresses and strains (Supplementary Materials Section S6). It is proposed that the observations from the current investigation in relation to joint loading mechanics were mediated at least partially as a function of the increased running velocity that was shown in habitual minimal footwear users when wearing conventional footwear. Furthermore, as previous analyses have shown the vasti muscles to be the most prominent predictors of peak medial tibiofemoral loading across a range of movements, it is also likely that this observation was instigated by the corresponding kinetic increases in this muscle group [58]. This observation suggests that in habitual minimal footwear users, conventional running shoes increase the risk from the mechanical parameters linked to the aetiology of medial knee OA [59,60,61] compared to minimal footwear.

Importantly and again in opposition to our hypotheses, this study showed using probabilistic modelling that medial tibiofemoral cartilage failure probability was statistically increased in conventional footwear in both habitual and non-habitual minimal footwear user groups. Although, with the exception of peak strain, the observed differences were below the MDC, in the habitual minimal footwear group it appears that the increases in failure probability were mediated as a function of the greater cartilage loading mechanics that were observed whilst running in conventional footwear. Interestingly, this was the case despite the decreased stride length and increased cadence in minimal footwear, and thus greater number of required footfalls necessary to complete the daily modelled running distance. However, in the non-habitual minimal footwear group, it was revealed that no significant increases in cartilage loading per footfall were observed, and the differences were all below the MDC indices. Nonetheless, when taking into account the duration (i.e., 37 years) over which failure probability was modelled, this investigation appears to show that the slightly increased cartilage loading (over several thousand loading cycles per day) in conventional footwear was ultimately sufficient to predict significantly greater cartilage failure probability in the conventional footwear. Therefore, the findings from the current study show that in both groups, running in the tested conventional footwear leads to a far greater probability of medial tibiofemoral OA in comparison to the experimental minimal footwear [14]. Tibiofemoral OA is characterized by debilitating and painful presentation [9], as well as long-term fiscal healthcare implications [7]. This investigation therefore shows that irrespective of whether runners are habituated or not, utilization of minimal footwear appears to be able to significantly attenuate runners’ likelihood of developing medial tibiofemoral compartment OA.

Sex is considered to be an independent risk factor for knee OA, and this pathology is more frequently observed in females [62]. It is not known whether our findings are generalizable to female runners, and it is therefore recommended that the effects of minimal and conventional footwear also be examined using probabilistic modelling in female runners. A further potential limitation to the current investigation is that the modelling of medial tibiofemoral contact strains and cartilage-failure probability indices were quantified using generic model parameters. The gravity of this drawback is less pronounced for the within-subject comparisons from this study, but may have a greater impact on between-subject analyses. Whilst the probabilistic approach to cartilage-failure modelling was itself designed to allow for the absence of subject-specific inputs, as the parameters (i.e., Power law and Weibull parameters) were derived from scattered experimental fatigue modelling data [50], there remains ambiguity in the specificity of cartilage structure, material properties and mechanical responses to imposed strains [22]. Whilst the modelling approaches adopted in the current study were necessary owing to the lack of scientific literature concerning cartilage responses to compressive loading [22], previous analyses have examined distinct approaches to fatigue loading using bespoke components of articular cartilage such as collagen fibers [63]. This would however necessitate a far more complex and computationally heavy modelling approach to translate gait and running analyses into bespoke strains experienced by specific cartilage structures, such that such approaches are likely not conceivable as broader research tools at the current time. The present approach to longitudinal cartilage-failure modelling is potentially appealing as only standard biomechanical data collection and processing indices are required, in addition to the computer code. However, it is not able to quantify loading and failure at bespoke macroscopic cartilage components, and other cartilage damage and failure models are in existence with more mechanical complexity [64], yet without the facility for movement-specific cartilage-compressive loading inputs. The most effective approach by which to address the research aims outlined in this study would be via a longitudinal prospective investigation, examining runners over the modelled time course (i.e., 18–55 years). However, prospective experiments on diseases with such longitudinal time courses as OA are rarely undertaken, owing to logistical and financial challenges. Overall, taking into account the experimental running volume and velocity, the musculoskeletal simulation and modelling approaches from the present investigation produced similar compressive joint loads and cartilage strains to both in vivo and previous analyses [19,22,52,53,54] as well as medial tibiofemoral failure probabilities to incidence rates of medial knee OA in both non-running and running populations [55,56,57]. However, it is important to recognize that this model is uncorroborated from the standpoint of predicting medial knee OA, and despite the difficulties associated with longitudinal model validation, this must be addressed in future computational and epidemiological analyses before definitive conclusions can be drawn from the current investigation.

## 5. Conclusions

In conclusion, no comparison of conventional and minimal footwear has previously been made in runners who habitually and non-habitually utilize minimal footwear, using a cumulative musculoskeletal simulation and probabilistic modelling approach of medial tibiofemoral cartilage. This investigation therefore augments current clinical and scientific knowledge in footwear biomechanics by examining the effects of minimal and conventional footwear on medial tibiofemoral cartilage failure probability in habitual and non-habitual minimal footwear users. In the habitual minimal footwear group medial tibiofemoral cartilage force, stress and strain were increased statistically in conventional footwear. Furthermore, in both habitual and non-habitual minimal footwear groups, cartilage failure probability was statistically increased in the conventional compared to the minimal footwear. The current investigation importantly shows that compared to running in minimal footwear, conventional footwear appears to have a negative influence on medial tibiofemoral cartilage health.

## Figures and Tables

**Table 1:** Experimental footwear characteristics.

	Minimal	Conventional
Mass (g)	167	285
Heel thickness (mm)	7	25
Heel–toe drop (mm)	0	14
Esculier et al. [23] minimalist index	92	20

**Table 2:** Participant characteristics.

	Non-Habitual		Habitual	
	Mean	SD	Mean	SD
Age (years)	26.15	4.74	27.00	3.26
Mass (kg)	68.54	5.32	67.77	6.19
Stature (m)	1.78	0.08	1.77	0.07
BMI (kg/m^2^)	21.86	2.50	21.81	2.93
Weekly running volume (km)	45.94	6.56	43.79	4.80
Duration of habitual minimal footwear utilization (months)			45.80	9.28

**Table 6:** Medial tibiofemoral cartilage failure probabilistic parameters.

	Non-Habitual				Habitual				
	Minimal		Conventional		Minimal		Conventional		
	Mean	SD	Mean	SD	Mean	SD	Mean	SD	
Probability of failure with repair (%)	33.18	29.48	47.19	31.71	32.81	32.08	50.00	34.16	**A, B**

Notes: **A** = significant difference between minimal and conventional footwear in habitual group. **B** = significant difference between minimal and conventional footwear in non-habitual group.

## Data Availability

Not applicable.

## Supplementary Materials

The following supporting information can be downloaded at: https://www.mdpi.com/article/10.3390/jcm11247335/s1. Supplementary Materials Section S1: Reliability and MDC values; Supplementary Materials Section S2: Sensitivity analysis; Supplementary Materials Section S3: Muscle forces; Supplementary Materials Section S4: Walking data; Supplementary Materials Section S5: Lateral tibiofemoral forces; Supplementary Materials Section S6: Regression analyses and comparisons between footwear with running velocity included as a co-variate.
