# Supplementary material for: Effects of Running in Minimal and Conventional Footwear on Medial Tibiofemoral Cartilage Failure Probability in Habitual and Non-Habitual Users
Source: J Clin Med. 2022 Dec 9;11(24):7335. doi: 10.3390/jcm11247335 (PMC9788348; doi:10.3390/jcm11247335)
Supplement: Supplementary file 1 [file jcm-11-07335-s001.zip › jcm-1994267-supplementary.pdf]

Section S1.

1. Reliability and MDC values

Sample: 10 male participants

	Walk		Run	
	**ICC**	**MDC**	**ICC**	**MDC**
Peak medial tibiofemoral force (BW)	0.951	0.27	0.901	0.61
Peak medial tibiofemoral stress (MPa)	0.947	0.01	0.899	0.02
Peak medial tibiofemoral strain	0.945	0.16	0.898	0.35

Table S1: Reliability and MDC values for running and walking.

	**Mean**	SD
Peak medial tibiofemoral force walking (BW)	3.25	0.55
Peak medial tibiofemoral force running (BW)	8.11	1.09

Note: MDC = 1.96 x SEM x square root of 2.

Section S2.

1. Sensitivity analysis

Sample: N = 10 male participants

Table S2: Walking and running velocities

	**Mean**	SD
Walking Velocity (m/s)	1.62	0.21
Running velocity (m/s)	4.11	0.44

Table S2: Input peak medial tibiofemoral forces during walking and running

Sensitivity analyses – linear line of best fit included to highlight the nature of the data distribution.

Anterior femoral arc in sagittal plane

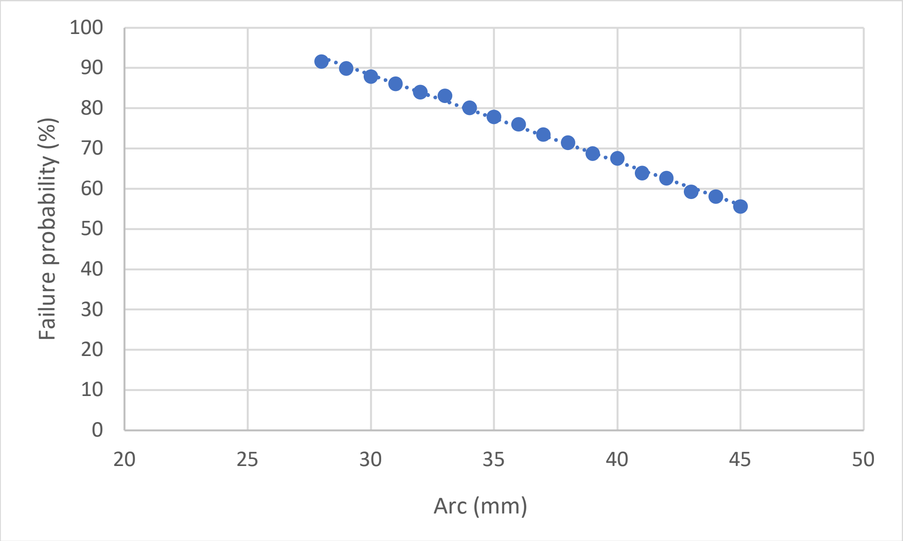
Failure probability (%)
100
90
80
70
60
50
40
30
20
10
0
20
25
30
35
40
45
50
Arc (mm)

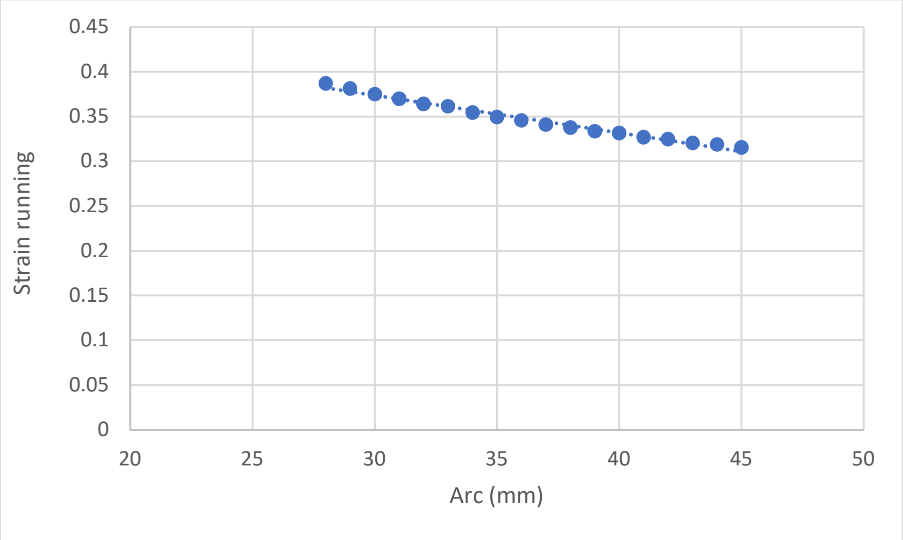
Strain running
0.45
0.4
0.35
0.3
0.25
0.2
0.15
0.1
0.05
0
20
25
30
35
40
45
50
Arc (mm)

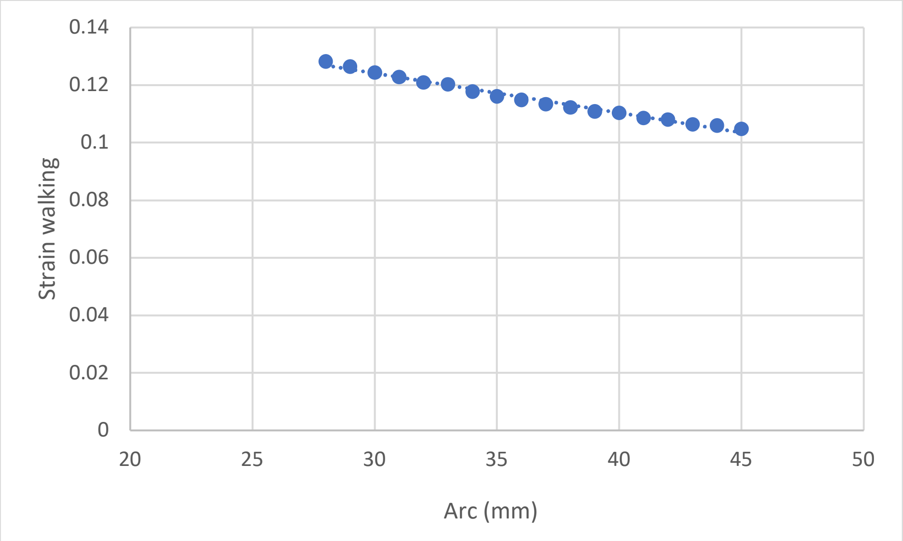
Strain walking
0.14
0.12
0.1
0.08
0.06
0.04
0.02
0
20
25
30
35
40
45
50
Arc (mm)

Femoral cartilage modulus

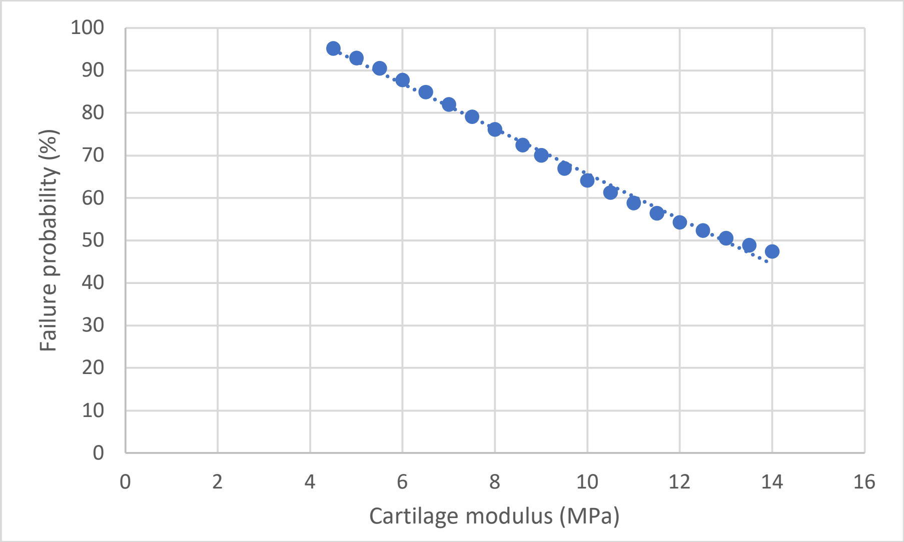

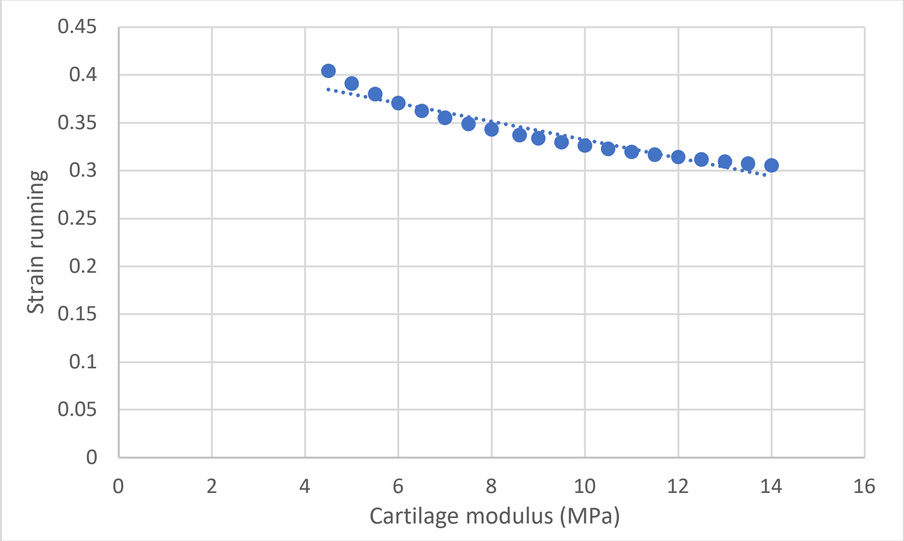

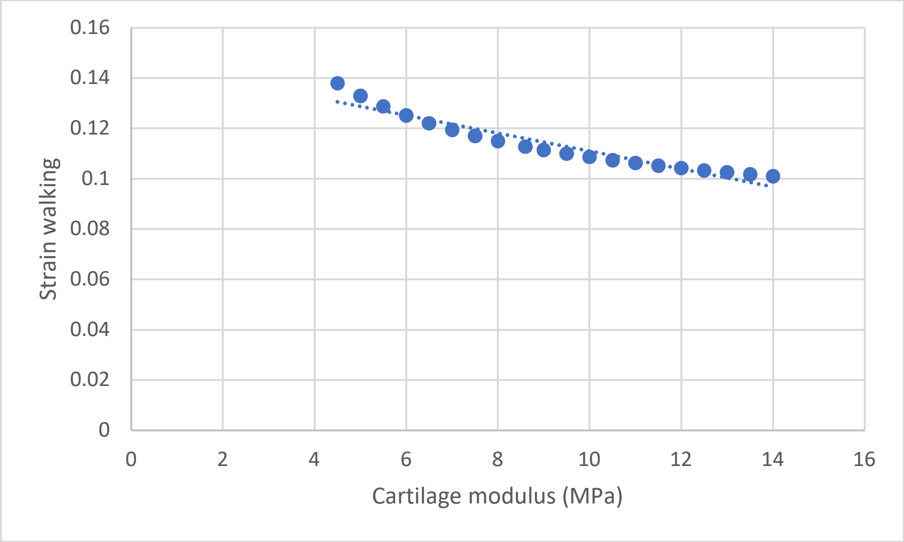

Covered tibial cartilage modulus

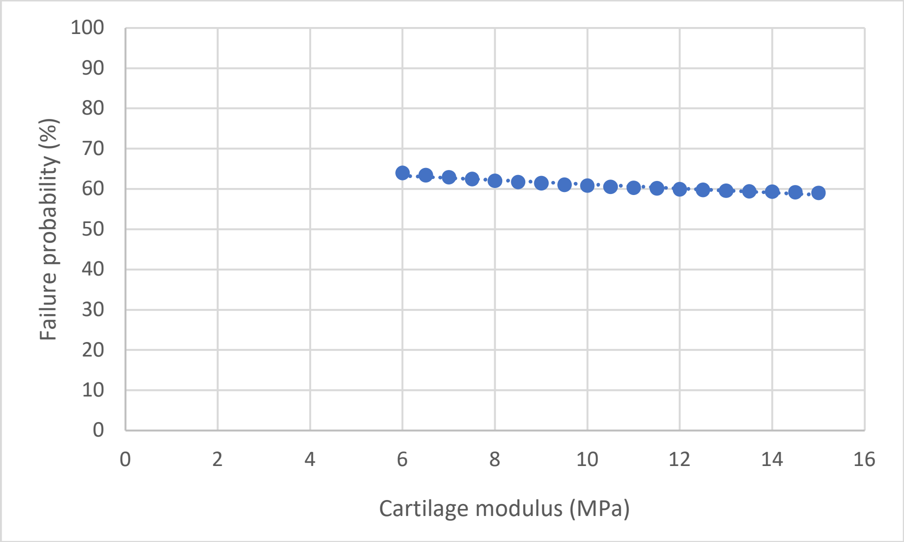

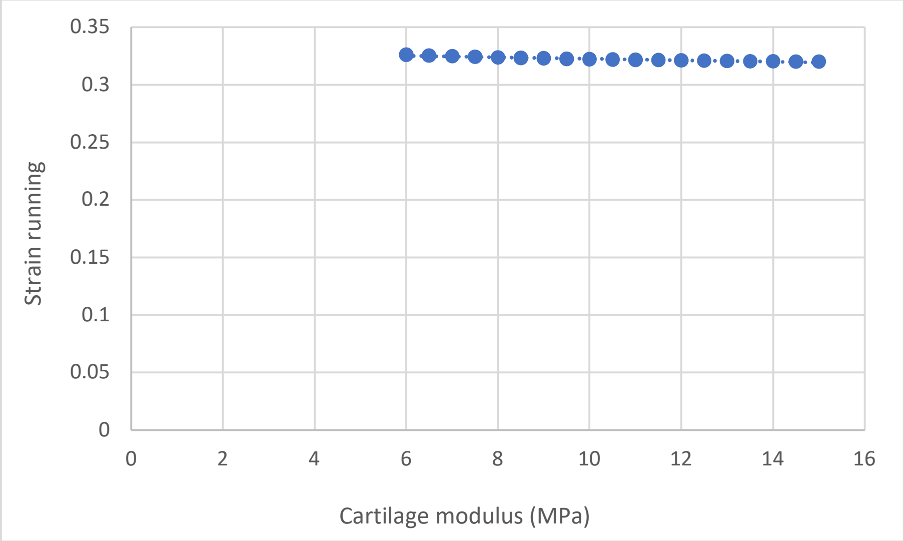

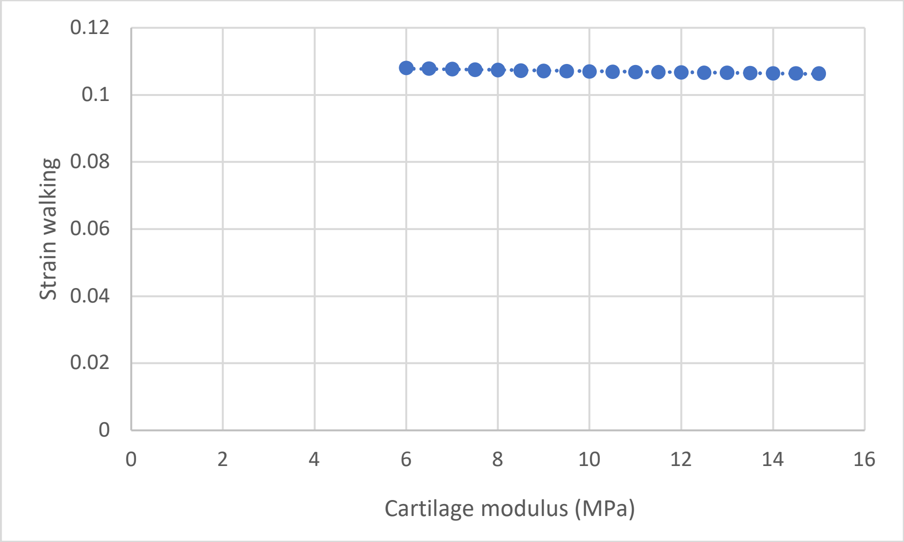

Uncovered tibial cartilage modulus

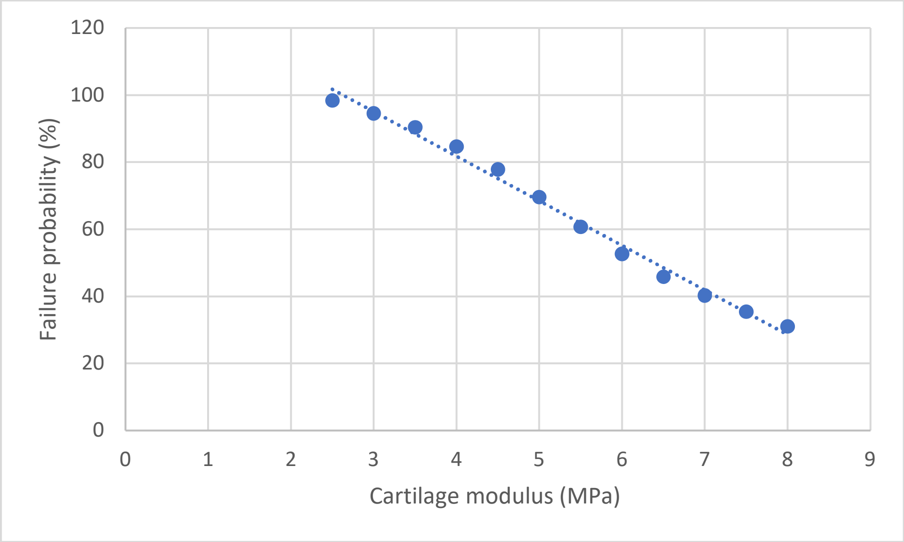

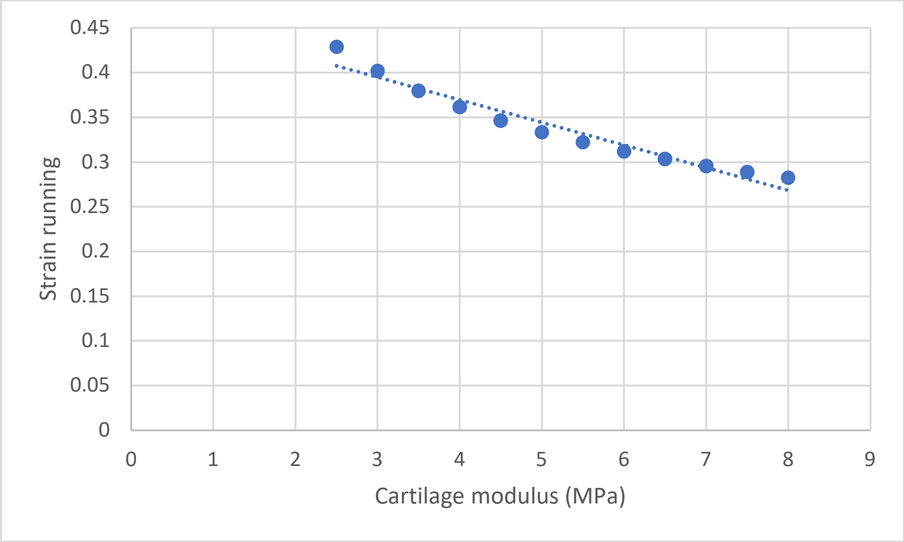

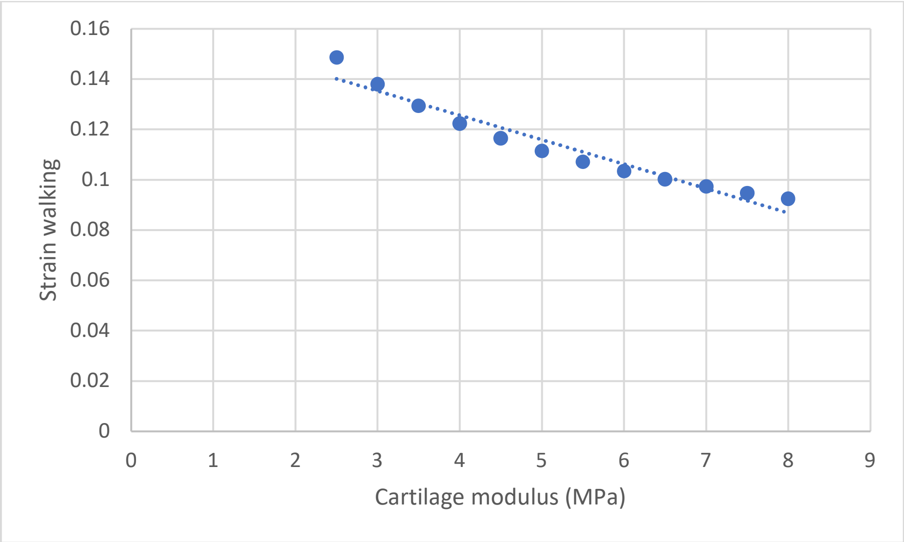

Meniscus modulus

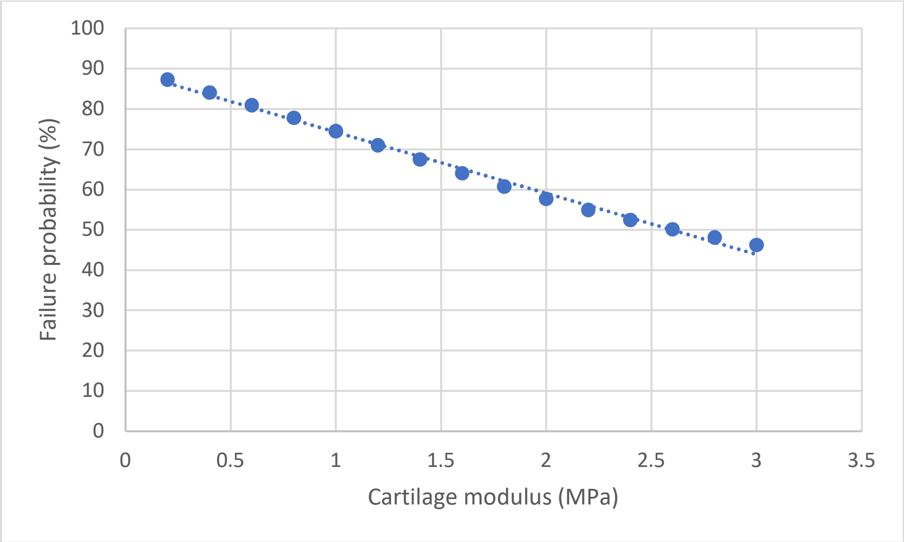

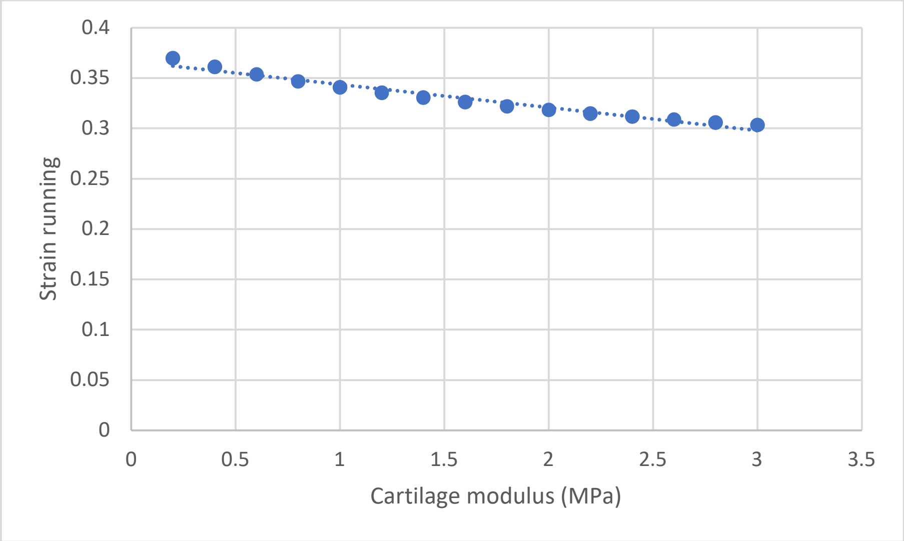

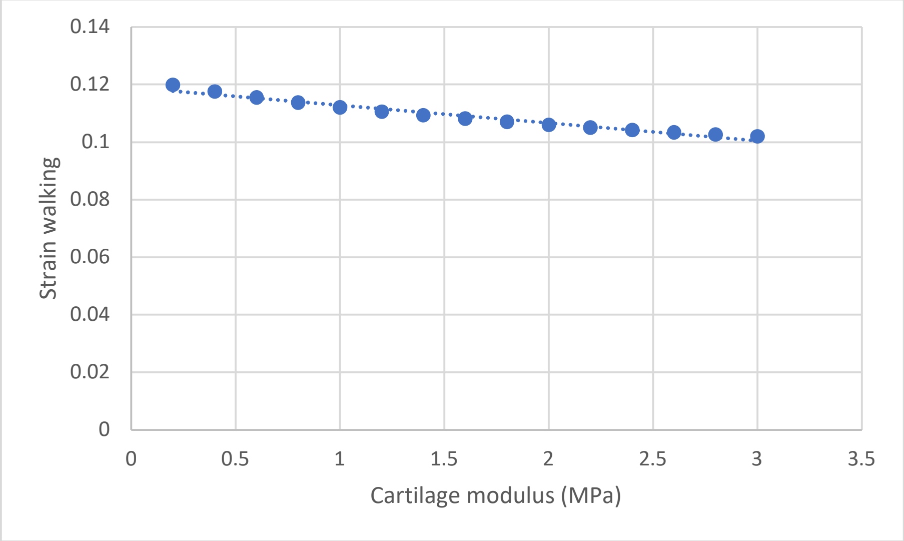

<u>Unloaded cartilage height</u>

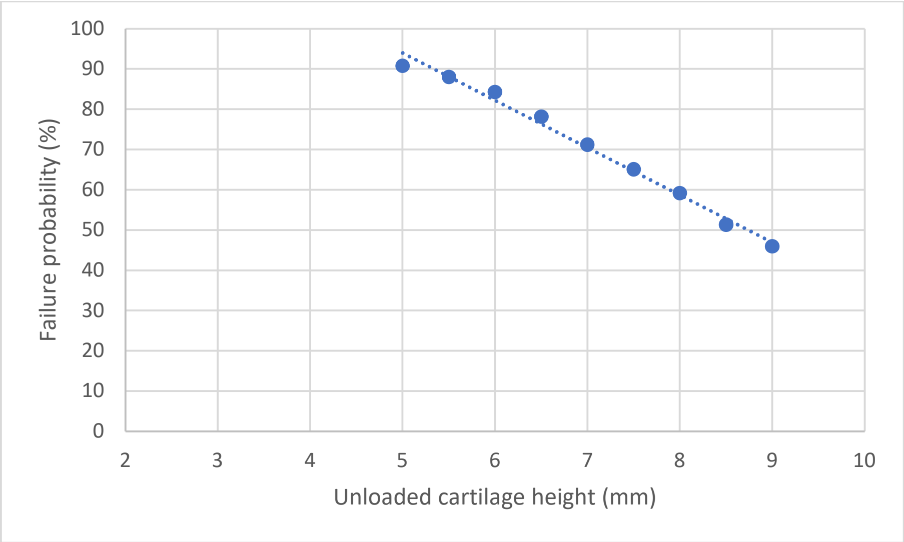
Failure probability (%)
100
90
80
70
60
50
40
30
20
10
0
2 3 4 5 6 7 8 9 10
Unloaded cartilage height (mm)

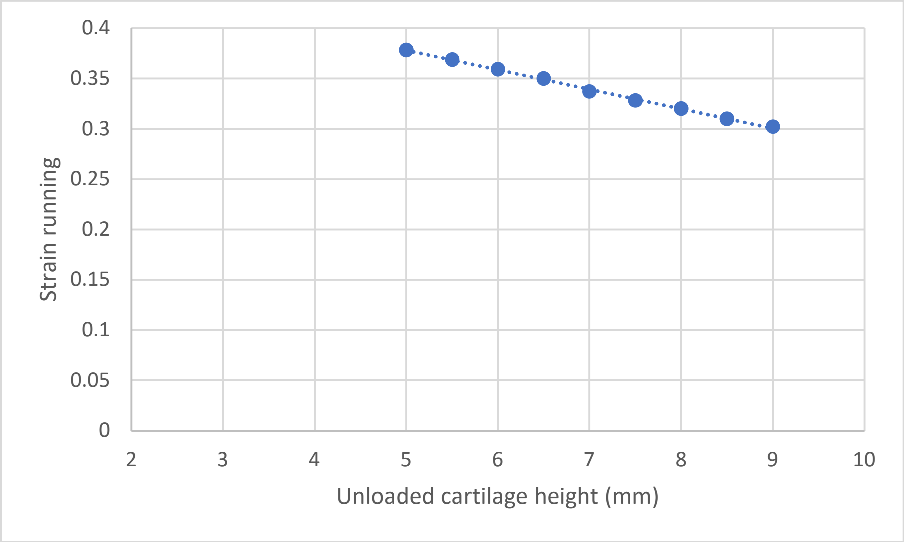
Strain running
0.4
0.35
0.3
0.25
0.2
0.15
0.1
0.05
0
2 3 4 5 6 7 8 9 10
Unloaded cartilage height (mm)

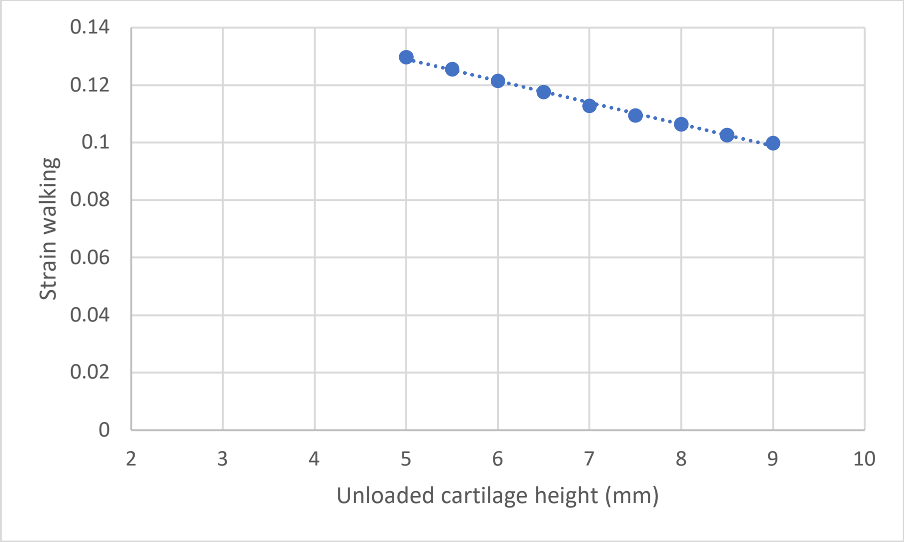
Strain walking
0.14
0.12
0.1
0.08
0.06
0.04
0.02
0
2 3 4 5 6 7 8 9 10
Unloaded cartilage height (mm)

Frontal tibial arc

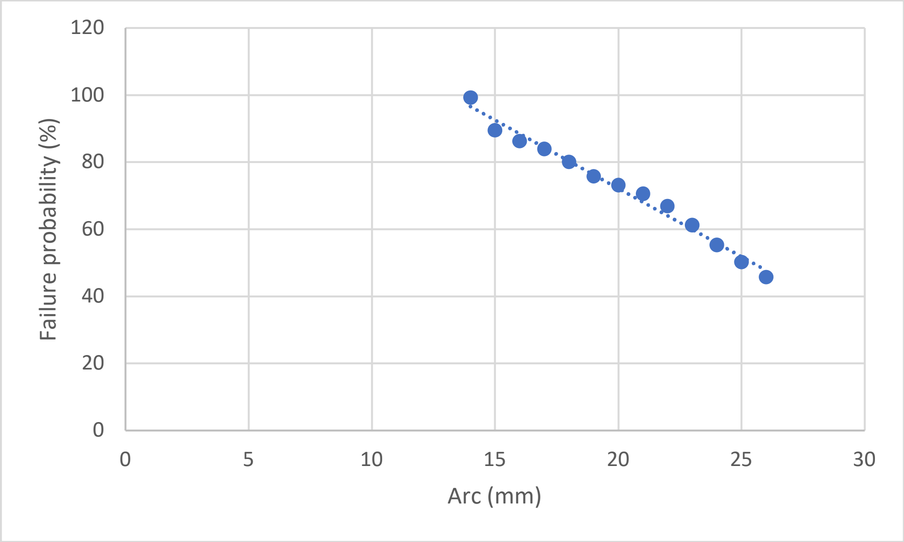

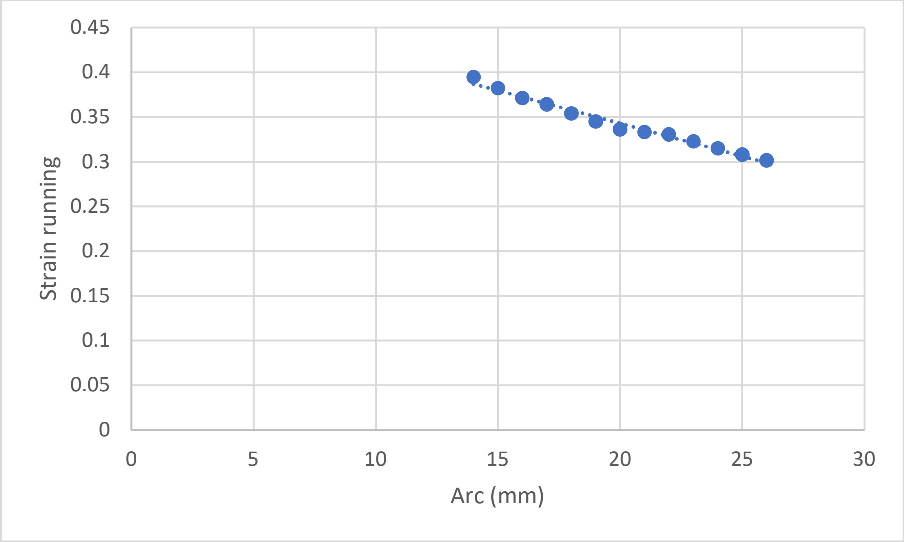

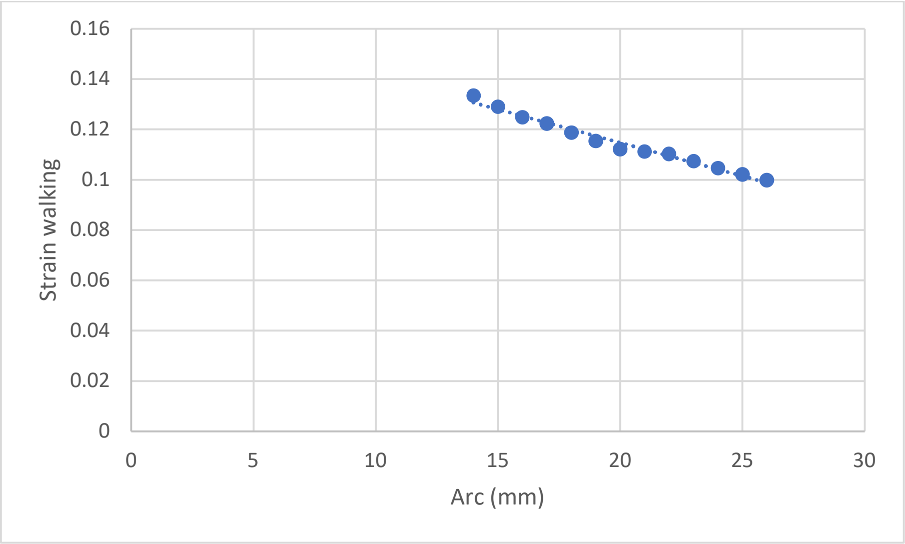

Poisson's ratio

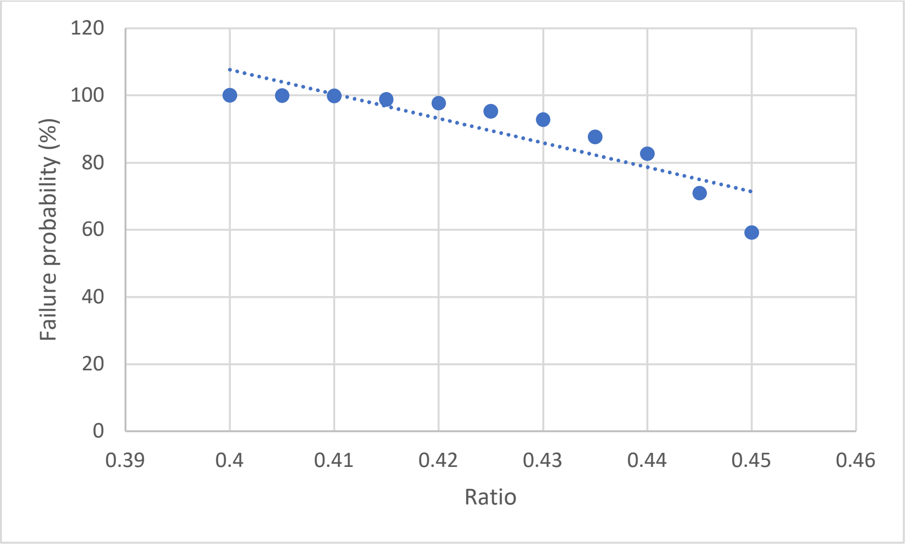

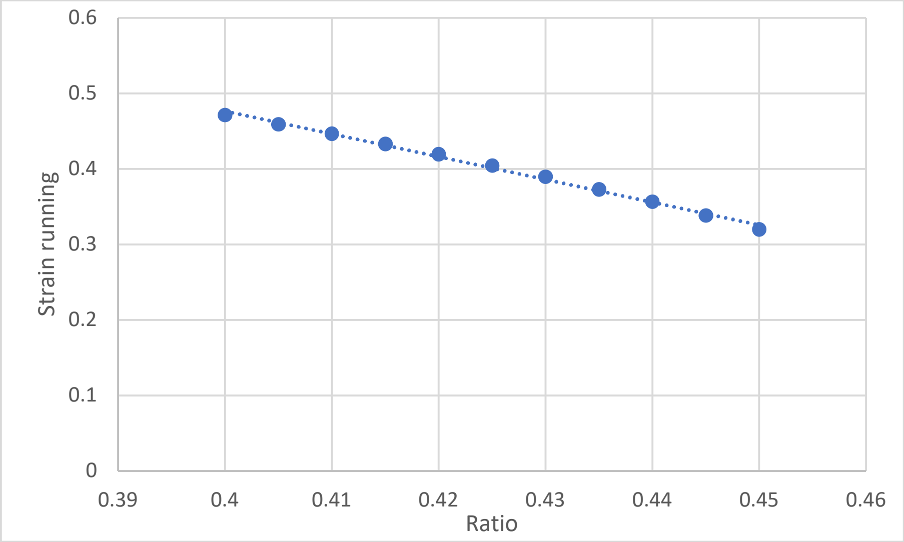

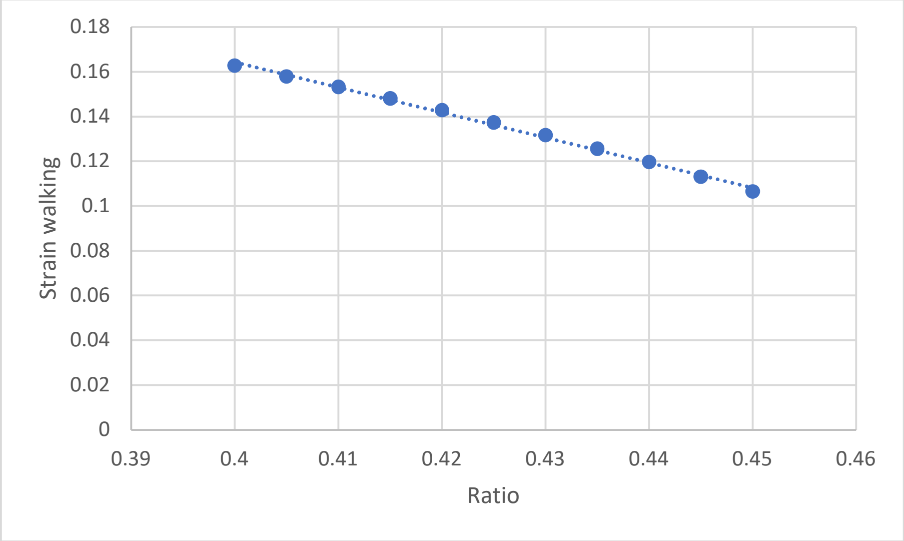

Section S3.

1. Muscle forces

Table S3: Muscle force parameters during running.

	**Non-habitual**				**Habitual**			
	**Minimal**		**Conventional**		**Minimal**		**Conventional**	
	**Mean**	SD	**Mean**	SD	**Mean**	SD	**Mean**	SD
Peak biceps femoris long head force (BW)	0.43	0.14	0.49	0.17	0.39	0.08	0.40	0.13

Biceps femoris long head impulse (BW·ms)	18.01	10.01	26.32	12.03	11.34	7.69	15.37	14.10
Peak biceps femoris short-head force (BW)	0.94	0.22	0.84	0.25	0.79	0.20	0.68	0.19
Biceps femoris short head impulse (BW·ms)	40.59	10.50	33.96	12.09	31.04	13.30	25.47	11.70
Peak gracilis force (BW)	0.10	0.04	0.07	0.03	0.08	0.04	0.08	0.04
Gracilis impulse (BW·ms)	2.65	1.15	2.36	1.07	2.12	0.69	2.06	0.68
Peak lateral gastrocnemius force (BW)	1.09	0.17	0.99	0.14	0.97	0.13	0.88	0.18
Lateral gastrocnemius impulse (BW·ms)	69.54	16.20	55.00	7.89	68.54	8.96	57.66	10.62
Peak medial gastrocnemius force (BW)	2.44	0.31	2.38	0.36	2.38	0.41	2.20	0.49
Medial gastrocnemius impulse (BW·ms)	156.85	31.18	140.58	23.50	182.32	24.57	156.33	33.96
Peak rectus femoris force (BW)	2.41	0.22	2.39	0.11	2.29	0.28	2.36	0.39
Rectus femoris impulse (BW·ms)	218.74	34.92	234.35	44.06	236.79	46.45	230.47	68.35
Peak sartorius force (BW)	0.29	0.02	0.28	0.03	0.26	0.03	0.25	0.04
Sartorius impulse (BW·ms)	20.92	5.45	19.73	6.29	17.57	4.79	14.87	5.44
Peak semimembranosus force (BW)	0.59	0.16	0.59	0.17	0.59	0.12	0.62	0.13
Semimembranosus impulse (BW·ms)	33.71	9.38	39.75	14.83	35.25	12.09	34.47	18.82
Peak semitendinosus force (BW)	0.27	0.08	0.25	0.09	0.24	0.09	0.25	0.06
Semitendinosus impulse (BW·ms)	12.99	7.78	13.23	7.18	11.21	9.36	12.11	9.18
Peak vastus intermedius force (BW)	1.93	0.31	2.02	0.34	1.89	0.49	1.99	0.59
Vastus intermedius impulse (BW·ms)	180.22	25.44	196.07	34.69	177.27	40.47	182.57	50.00
Peak vastus lateralis force (BW)	3.08	0.36	3.24	0.41	2.98	0.64	3.11	0.83
Vastus lateralis impulse (BW·ms)	279.11	40.94	308.05	55.48	271.50	60.65	280.86	72.95
Peak vastus medialis force (BW)	1.72	0.28	1.82	0.33	1.74	0.46	1.83	0.54
Peak vastus medialis impulse (BW·ms)	161.51	23.10	175.44	31.22	160.56	37.28	165.99	46.01

In the habitual minimal footwear group, biceps femoris short head impulse (b = 5.57 (95% CI = 3.34 – 7.80), t = 5.23, P<0.001), peak biceps femoris short head force (b = 0.10 (95% CI = 0.05 – 0.16), t = 3.85, P=0.001), lateral gastrocnemius

impulse (b = 10.88 (95% CI = 4.53 – 17.22), t = 3.59, P=0.002), peak lateral gastrocnemius force (b = 0.09 (95% CI = 0.03 – 0.15), t = 2.97, P=0.008), medial gastrocnemius impulse (b = 25.99 (95% CI = 9.43 – 42.56), t = 3.28, P=0.004), peak medial gastrocnemius force (b = 0.18 (95% CI = 0.05 – 0.31), t = 2.86, P=0.01), sartorius impulse (b = 2.70 (95% CI = 1.63 – 3.78), t = 5.26, P<0.001) and peak sartorius force (b = 0.01 (95% CI = 0.001 – 0.03), t = 2.33, P=0.031) were significantly greater in the minimal in relation to conventional footwear. However, in the same group, peak vastus medialis force was significantly larger (b = 0.10 (95% CI = 0.01 – 0.20), t = 2.42, P=0.039) in the conventional compared to minimal footwear (Table S3).

In the non-habitual minimal footwear group, biceps femoris short head impulse (b = 6.63 (95% CI = 1.38 – 11.87), t = 2.64, P=0.016), peak biceps femoris short head force (b = 0.10 (95% CI = 0.04 – 0.16), t = 3.39, P=0.003), peak gracillis force (b = 0.03 (95% CI = 0.002 – 0.05), t = 2.32, P=0.032), lateral gastrocnemius impulse (b = 14.54 (95% CI = 9.31 – 19.78), t = 5.82, P<0.001) and peak lateral gastrocnemius force (b = 0.10 (95% CI = 0.01 – 0.19), t = 2.44, P=0.025) were significantly greater in the minimal in relation to conventional footwear. However, biceps femoris long head impulse (b = 8.31 (95% CI = 3.81 – 12.80), t = 3.87, P=0.001), rectus femoris impulse (b = 15.61 (95% CI = 2.45 – 28.78), t = 2.48, P=0.023), semimembranosus impulse (b = 6.04 (95% CI = 2.05 – 10.04), t = 3.17, P=0.005), vastus lateralis impulse (b = 28.94 (95% CI = 4.30 – 53.59), t = 2.46, P=0.024), vastus medialis impulse (b = 13.93 (95% CI = 0.51 – 28.37), t = 2.40, P=0.048) and vastus intermedius impulse (b = 15.85 (95% CI = 0.14 – 31.84), t = 2.51, P=0.022) were significantly in the conventional compared to minimal footwear (Table S3).

Section S4.

1. Spatiotemporal variables

Table S4: Spatiotemporal variables during walking.

	**Non-habitual**		**Habitual**	
	**Mean**	SD	**Mean**	SD
Velocity (m/s)	1.45	0.14	1.58	0.19
Stride length (m)	1.52	0.08	1.58	0.18

Walking velocity was found to be significantly faster in the habitual minimal footwear group (b = 0.13 (95% CI = 0.03 – 0.24), t = 2.52, P=0.016) (Table S4).

2. Joint contact forces

Table S5: Joint contact force parameters during walking.

	**Non-habitual**		**Habitual**	
	**Mean**	SD	**Mean**	SD
Peak medial tibiofemoral force (BW)	2.85	0.54	3.05	0.53

Medial tibiofemoral cumulative load (BW/m)	1.34	0.23	1.27	0.22

No significant differences in medial tibiofemoral forces were observed between groups (Table S5).

3. Stress/ strain

Table S6: Stress/ strain parameters during walking.

	**Non-habitual**		**Habitual**	
	**Mean**	SD	**Mean**	SD
Peak medial tibiofemoral stress (MPa)	2.22	0.53	2.35	0.34
Peak medial tibiofemoral strain	0.15	0.03	0.15	0.02

	**Non-habitual**				**Habitual**				
	**Minimal**		**Conventional**		**Minimal**		**Conventional**		
	**Mean**	SD	**Mean**	SD	**Mean**	SD	**Mean**	SD	
Peak lateral tibiofemoral force (BW)	4.38	0.98	4.00	0.55	3.80	0.72	3.74	0.69	***B***

No significant differences in medial tibiofemoral contact mechanics were observed between groups (Table S6).

Section S5.

1. Lateral tibiofemoral forces

	**R^2**	**P**
Peak medial tibiofemoral force (BW)	0.262	<0.001
Peak medial tibiofemoral stress (MPa)	0.152	0.01
Peak medial tibiofemoral strain	0.141	0.02

Table S7: Lateral joint contact force parameters

Notes: B = significant difference between minimal and conventional footwear in non-habitual group.

Section S6.

1. Regression analyses.

Table S7: Regression coefficient of determination values with running velocity as predictor.

2. Comparisons between footwear with running velocity included as a co-variate.

Habitual minimal footwear users

Peak medial tibiofemoral force (*b* = 0.23 (95% CI = -0.63 – 1.11), t = 0.89, P=0.581) was not significantly different between the conventional and minimal footwear. However, peak stress (*b* = 0.48 (95% CI = 0.06 – 0.95), t = 1.98, P=0.048) and strain (*b* = 0.02 (95% CI = 0.003 – 0.04), t = 1.99, P=0.047) were significantly greater in conventional footwear.

Non-habitual minimal footwear users

There were no differences in peak medial tibiofemoral force (*b* = 0.15 (95% CI = -0.39 – 0.69), t = 0.55, P=0.59), peak strain (*b* = 0.27 (95% CI = -0.13 – 0.67), t = 1.37, P=0.18) or peak stress (*b* = 0.01 (95% CI = -0.006 – 0.03), t = 1.34, P=0.19) between minimal or conventional footwear.
